# Points of Interest in Oral Surgery

**Published:** 1912-12-15

**Authors:** J. A. Gardner

**Affiliations:** Memphis, Tenn.


### POINTS OF INTEREST IN ORAL SURGERY.

BY J. A. GARDNER, D.D.S., MEMPHIS, TENN.

Read before the Tennessee Dental Association, June 8, 1912.

In recent years my attention has been especially directed to oral sugery. I believe that as a profession we are more deficient in the medical and surgical sides of our profession, than we are in the mechanical, and to my mind, the surgical affords a greater field of opportunities for the dentist to accomplish better things for his patients, than in any other branch of dentistry.

A thorough knowledge of the pathological conditions which are met with so often in the mouth, makes it possible for us to render invaluable service to our patients in so many cases. How many lives have been lost by that dreadful disease, cancer, and how many of those lives could have been at least, prolonged, had they been properly treated in the beginning, and as there are so many of these growths or tumors in the oral cavity which are benign in their beginning, and later, take on a malignancy which terminates the life of the patient. It behooves us, as dentists, to prepare ourselves that we may be able to detect these conditions at a glance. Only recently a lady came to me for dental services, and the first thing my vision met, was a typical epithelioma of the tongue. It had progressed to the stage of the lancinating pains unnoticed, but fortunately for her, it had not reached the lymphatic glands. The tumor was about the size of a chestnut and by a v-shaped incision it was removed with little difficulty. The edges were coapted with cat-gut and the case was well in a few days. A specimen under the microscope revealed the typical flat coll of the epithelioma.

Many cases of chancre and secondary lesions of syphilis escape the notice of the dentist, which involves the safety of his patients and the operator himself. If such conditions could be detected how many dentists would be able to positively diagnose them and unhesitatingly give the treatment necessary. I refer to syphilis because of the certainty of its diagnostic signs, and the simplicity of the treatment. I venture to say, that not ten per cent of the practicing dentists would be willing to rely on their own judgment in the diagnosis and treatment of syphilis manifestations in the mouth, and yet, there is no question as to the D. D. S. authority for treating systematically or surgically for the cure of any disease or condition presenting itself in the mouth, if he be competent.

How many graduate dentists are there in our profession that would not quiver in his boots, even at the thought of performing an operation for cleft palate, or for the removal of an osteo-sarcoma, epithelioma, multi-locular, dentigrous, or any other tumor or cyst, presenting within the mouth or upon the jaw—the field upon which we have planted our flag and to which we lay claim as masters? And yet, Gentlemen, when a serious problem arises in this field, in which we claim to have specialized, which involves life or death or deformity for life, we hurry to the medical practitioner for diagnosis and treatment, or to the general surgeon for an operation, instead of treating ourselves or consulting with a better qualified brother dentist.

Is it not up to us as specialists upon the mouth and jaws to become more capable than the medical practitioner or general surgeon in our field? Rather, *they* should be the ones seeking advice in consultation with us.

I hear so many dentists criticising the medical profession, and the general public, because they do not place the dental profession upon a higher plane. Gentlemen, we have no other shoulders upon which we can rightly place the blame for these conditions, save our own. If we as a profession, are masters of all we survey and are able to take care of the most critical conditions which may arise in our domain, then, and then only, will the medical profession give us the right hand of fellowship, and the layman regard us in the light which we desire, and which we will then deserve.

The highest honor to which man can attain is that of the ministry, whose work it is to assist in the salvation of souls, and if possible, point out through the gloom of sorrow's night, the stars that shine beyond. The next highest honor to which man can aspire is that of the Doctor, whose duty it is to assist in the salvation of the physical man, and that branch of medicine which deals with the life destroying diseases, or that can change a death warrant into health and happiness deserve the highest honor. And, Gentlemen, the mouth and jaws are liable to many diseases which threaten the health and often the life of the people.

I was recently called to see a little girl six years old, suffering from noma of the cheek, following a case of scarlet fever. The patient was in a very serious condition and all hope of life had been abandoned. Remembering the report of a case in which 10 c.c. of anti-streptococus serum was administered and the patient recovered, I advised the same treatment in this case, and the little patient recovered. There are very few cases of noma which do not prove fatal, and there are many other diseases which affect the mouth and are most serious, and for some, little is known of a rational and specific treatment. Therefore, it behooves us, as specialists in this field, to study and develop the medical and surgical side of our profession. We have heretofore made wonderful advances in the technical, but we have woefully neglected the medical and surgical treatment of the mouth. When we review the past, and find the Horace Wells, a dentist discovered the anesthetic effect of nitrous oxide gas, and Lemonnier, a French dentist, was the first to suggest and successfully operate for the closure of fissures of the palate by surgical operation, it seems to me that we are going backward instead of forward. This record was published in 1776. Lemonnier cucceeded in closing a fissure in both the hard and soft palates by paring the edges of the cleft with a knife and approximating them by the use of sutures.

It appears to me that since this operation for cleft palate, the most difficult of all oral surgery was first suggested and performed by one of our profession, and because of the peculiar familiarity of the dentist with the mouth and jaws, the dentist is the logical man to do the surgery of the mouth, and I would suggest that more stress be placed upon the teaching of this subject in the dental schools. We have entering our dental colleges every year, just as good material to make oral surgeons, or rather to lay the foundation for an oral surgeon, as we have entering the medical colleges, out of which they succeed in laying the foundation for a general surgeon, therefore, we as a profession, should demand the proper course of instruction, in our dental colleges, which will prepare the dental graduate to do oral surgery. What should we think of the medical doctor, that called himself any part of a surgeon, who hesitates to perform any operation wherein the patient would have a better chance for life by virtue of the operation, just because it would involve a greater responsibility on his part, as a surgeon to undertake the operation.

We have both general and oral surgery taught in our dental schools, but if we are not expected as dentists to put into practice by the actual doing of oral surgery, why should we teach it in our schools, any more than in schools of pharmacy, or to the optician or even the manicurist. I do not think it wise to lay claim to anything in which we can not deliver the goods, but in my opinion, we can deliver the goods in oral surgery far better than the general surgeon, if we will only educate,our minds and develop our skill in that direction.

Where would the dentist, or general surgeon, or layman refer a patient with an alarming condition of the eye? Why, to the eye specialist, of course And yet, the eye specialist sometimes repairs or constructs a pair of glasses, or he inserts a glass eye, and yet, he is not referred to as a mechanic, but he enjoys as lofty a position as any other man in the medical world. And why? Simply because he does not shirk the responsibility of the most serious operation in his field.

Just the other day one of our foremost dentists referred a patient to a general surgeon for a simple frenum-labium operation, for which the surgeon charged a fee of fifty dollars. Another case was referred to a general surgeon, by her dentist, who was afterward referred to me by the surgeon. It was simply a case where the cheek tissues through an inflammatory process, had become adherent to the ridge on the left side of the lower jaw, at a point corresponding to the second bicuspid and molar.

It is not the purpose of the writer to lessen the interest in the mechanical side of our profession, but to increase the interest in the surgical side. However, if it is the consensus of opinions in the profession that a dentist should not attempt the surgery of the mouth and jaws, without holding the M. D. degree, but should confine himself to the teeth, the pulps of teeth, and the immediate soft tissues, wherein is not involved life or death, or a serious deformity, or which does not require unusual knowledge in medicine and surgery, then I am strong for leaving the surgical branch out of the curriculum of our dental schools, and of allowing the student to devote his entire time to the work he is expected to do as a dentist, eliminating the idea that is so prevalent among dentists, that they should expect to hold the same exalted position with the man that deals with the problem of life and death in the medical world.

Gentlemen, with these facts before you, claiming as we do, to be fellow members of the great healing craft, aside from our standing in the medical world, are we doing our duty?

DISCUSSION.

Dr. Ewell Neal: I have often heard the opinion expressed, that we should broaden out and get away from the mechanical side of our work. I took the position at one of our recent meetings that too much stress is being laid upon the mechanical side of our work and too little upon the fundamental side. When a dentist leaves college, he then has his life work before him, in which to acquire proficiency and to develop influential character. When he leaves college he leaves behind him the teachers, the books, and actual laboratories in which he studied while in college; he leaves behind all the fundamental things of the school and he does not have their advantages. He takes up the practical work in which the public is most interested and becomes absorbed in that department of the work. The doctor spoke of Noma and Cancer, one of the most deadly diseases known to medical science. I think it is a crime, at least it should be considered criminal negligence for a dentist to be unable to recognize a case of cancer.

In twenty-four hours a carcinoma may grow extensively, and unless the patient gets quick attention, in less than ten days, the undertaker takes charge of the patient. So you can see how important it is to take early action in some of those cases, and operate immediately, if the life of the patient is to be saved.

I had occasion in the latter part of the winter to see an interesting case of a little negro boy eight years of age. A negro physician called me over the telephone one day. He said he had something extremely interesting. He says, "I heard you lecture on oral diseases in our college about five years ago, and it seemed to me that you described this case. I would like you to see this case. I went with him and when I saw the child, the tumor had extended from the molar teeth almost down to the angle of the jaw, and had encroached upon the cavity of the mouth. It was a heart breaking picture to see, and if you once see a case it would cause you to take more interest in these conditions. It is a responsibility that rests upon the dentist to diagnose these cases in their incipiency.

There is nothing I can add to the paper in this connection, except to impress upon you the importance of a wider view of our work, and instead of limiting our view to the actual operation upon the teeth, when we look into the mouth, we should see more than the teeth; we should see every condition present, and be able to recognize those conditions that are threatening, and remember an early diagnosis in many cases is absolutely necessary if the life of the patient is to be saved. The only safe method of procedure is to take a specimen of the patient's case and get a microscopic section to study and verify your judgment and follow that up with an actual operation. A red hot poker may be the best thing you can use, burn and seer that part diseased, until you destroy every vestage of the disease. If the bone is implicated a thorough bone operation must be made.

And in regard to the ability of the dentist to recognize these conditions and in living up to the degree conferred upon us, instead of trying to find another degree or name that would better fit us, I certainly believe in fulfilling all our obligations. We must cease to be merely mechanics, and learn to be professional men in the broadest sense.

There is nothing I like more than to meet the progressive men of my profession; I learn something everytime I meet such men. There is no reason in the world why dentists should not do these operations if they know how; when you request the surgeon to do this work you have lost your name and you have lowered yourself in the esteem of the medical world. It should be the aim and desire in the hearts of the young men who are coming after us that they must know surgery, not just for the sake of practicing it or of having a license to practice it, but he must remember that this is to be a part of his life work, and he will need it, as he must apply it to his practical work later on; he must get away from the mechanical only, and be a conservative physician.

Dr. W. C. Gillespie: The essay was a spirited one, and is all right, and very necessary, at the same time, there are a few things to be said on the other side. It is undoubtedly true that many in our profession think that the only necessity laid upon the profession is to do no more than fill teeth and they will never do more than fill teeth; and it is just the same way with anything else. I do not want to call the Doctor an enthusiast, but the enthusiast is one of the most useful individuals that we can have around, for he stirs up the so-called conservative. Most of us would not know a cancer at all, if some one did not cite us to an ideal condition. I am not calling the essayist a fanatic, but he is filling that position in a most acceptable sense, as he is sincere and seems to believe in his views. Just like the medical Doctor, you have to believe that an operation is necessary, at the same time, it is not necessary for you to operate. You often see a medical man who devotes himself entirely to surgery, and as a surgeon he is not lowered in the esteem of his medical brethren; and this has not lowered him in the eyes of the people, because he leaves out the general practice and gives all of his attention to surgery. We have a number of doctors in Nashville, that do not attempt surgery at all, and it seems no one thinks any less of them. Some dentists think it is very necessary that Dental Surgery be taught in the school; at the same time every man who practices dentistry learns a good deal of it and he may soon become a good surgeon; that is, he may be fitted to perform surgical operations.

I have just recently had occasion to call in a surgeon to operate on two cases of mine. It is necessary for dentists to be able to recognize those conditions, and it is up to him to see that his patient gets the best service. He can not as a rule carry a complete outfit of surgical instruments, and be as ready and well equipped to do surgery as to perform an operation in dentistry only. Until you get proficient to that extent that you can diagnose the condition of your patient and recognize all his conditions, you should put him in the hands of the man best fitted and able to perform that operation. At the same time I do not want to excuse any one for his inability. I want to know that all my patients have the best service possible rendered them, and I seek out a man who I think is professionally able to perform that particular operation. We are in position to make a great many suggestions to the surgeon, and through the working together, and by our combined efforts, we give our patient a better operation, than either one could do singly.

If a man has natural ability to perform surgical dentistry, then he should devote the greater part of his time to surgical operations; otherwise he had better leave it alone.

Dr. E. V. Wood: The longer I live, the harder I try to do perfect work, and the right thing. I want to speak a little of the experience I have had, which is very limited, and I do not want to exalt myself, but as I stated, the longer I live, the harder I try to be perfect, to be more practical in everything I try to do. I want to get in a little issue, or word. Doctor Gillespie spoke of our limited knowledge of many things in oral surgery. I think it would be a good idea to delve deep into anything we have to do, at least to the extent of being proficient enough to take care of whomsoever has placed himself in our hands.

I do not know much about oral surgery, if you please. I know how to put on a gold crown, and how to extract the pulp of the tooth, and some other such things about the mouth and teeth, but I believe we should know how to do it as much as the fellow who has made it a special study, even though we may not be perfect in skill.

On last Christmas day, I was called from my home to the office to see a patient who was having trouble with the right side of the lower jaw. He did not know what it was, and I diagnosed the case as necrosis. I performed the usual operation, and got a piece of bone almost as large as my thumb. Then the patient got a little better, but later I was called on again to perform the same operation, and this operation was of the same character. My patient got a little better again, but the necrosis persisted. I did not feel competent to operate in major surgery, as I thought it might be necessary to take out the entire side of the jaw. So I told my patient that I was not skilled enough to perform such an operation to guarantee a recovery. If I had assumed a knowledge of oral surgery and had operated, and taking the chance of the life or death of my patient, it would have been criminal.

I say we should go so far as we know and can go and stop right there. We should continually add to our limited knowledge as we go along and put what little knowledge we gather into practice, but be sure we are right and then go ahead.

I must turn over that which I do not know how to do to some man who is more perfect in knowledge of the work. I can not delve into oral surgery momentarily and master the situation. If we are not willing to risk our own judgment, we should refer surgical cases to a surgeon. I am not satisfied with the knowledge or experience I have had to consent that I risk the life of my patient in my own hands. I think it would be dishonest for me to operate and experiment upon human beings.

Dr. Ewell Neal: Dr. Gillespie spoke of practitioners of medicine who decline to do surgery. We have some very efficient medical men who make no attempt to do surgery and refer all surgical operations to the surgeons. But when operations are necessary, these practitioners, when called upon and have to, they can do the work. The reason the dentist is looked down upon is not because he does not do this work, but because he does not know how to recognize the symptoms of these diseases. I do not believe that the dentists ought to do difficult oral surgery.

Dr. H. W. Morgan: Every now and then in our Association, and in our personal intercourse one of the subjects discussed in the paper, the recognition of the dentist as a professional man is introduced. In some sections it is a question of society standing in dentistry, while in the other, it is a question of their relation to another branch of medical knowledge.

I never hear the discussion that I do not think that we are making an error, and certainly not elevating ourselves. We are standing up a straw man to knock him down, and in so doing we are degrading the profession we love.

The ground of the paper, that the profession is not taking sufficient interest in medical learning, and that the incoming profession is being insufficiently schooled can not be substantiated. What the dentist considers as the medical side of his profession, in my judgment is a gross error, and one that contributes to a misunderstanding. It presumes that we are not entitled to a standing equal with the aggressive medical profession. I believe as never before the dental profession, and those who have to do with the training of the new profession are more keenly alive to the importance, not in surgery alone, but in everything in the way of knowledge and skill that makes for more efficient practitioner.

It is not alone the question of a man's ability to recognize the necessity of an operation, it is far deeper than that. Our teachers do all they can to elevate the profession by giving the students the broadest curriculum the time will permit, and God knows, if you can read it in the young men's faces, you would believe they were getting not only the science of oral surgery, pathology, etc., but a great deal too much of too many subjects

I can count with the fingers of my two hands all the great oral surgeons in the world; their professional work is marvelous and we must not deny it, and yet it is hardly a century since the building of the first dental college. If that is true, why should we deplore the fact that there are dentists who do not recognize surgical conditions, and who are not willing to undertake an operation when there is a question of failure or death to be taken into account.

It is not in my judgment a good idea to give voice to the world, that we know nothing of oral pathology or surgery. Permit me to make this statement, that everything a dentist does, no matter how trivial it may be, it is of a surgical nature, from the extraction of a tooth to the resection of a jaw.

Dr. J. L. Mewburn: I think many times we give up the case too soon. A few unusual cases require investigation, and because they are a little difficult, is no reason why we should refer them to the general surgeon. Usually every surgeon will say, "We will have to operate and scrape the bone, and that means a bill for $50.00 and another operation in a little while.

I will recite a case I had thirty-five years ago, and I had been out of college only three years. The case happened here in Memphis.

The father brought a girl eight years old to see me, to have a tooth examined. I found a tumor by her upper right cuspid. I did not know what it was, but a limited examination revealed it to be an excessive tumor. I made up my mind to use arsenic to take it out, but rather than take the risk of a child's life at that age, I called in five of our most prominent physicians and surgeons, and I sent a note that I had a very interesting tumor, and to be at my office next day at 10:00 o'clock. They were all present. I placed the child in the chair, and exposed the tumor, and told the physicians that I proposed to use arsenic to remove that tumor, and I says, "I have had you to come here to get your opinion." As soon as I said "Arsenic," all five physicians jumped on me with both feet. Said, "You do not know what you are talking about. You are taking the risk of the child's life." I says, "That is what I propose to do." They suggested that I remove the deciduous teeth. I says, "That is very reasonable, I will do it." So I extracted the teeth, and waited a few days, and as soon as the pressure was reduced, the tumor filled the mouth and was worse than before. The next suggestion was to puncture it with C. P. Nitric acid. I thought it best to call in the physician who advised Nitric acid treatment to assist. I punctured it through and through, using an orangewood stick. I never saw anything puff out like that did. It puffed up like a cauliflower. Then the next thing was to call in the man who advised the use of the three chlorides. I called him in, and I says, "What do you suggest?" He says, "Get me a piece of chamois." Well, I got the chamois. He says, "I can not put that in the child's mouth; it won't stay in the mouth." That left me with one other alternative of medical counsel, so I called in the next man, who was the surgeon. He says, "No use in talking, you have got to operate and take the tumor out down to the bone. It makes no difference what you do now, you have got to get it out." I saw the father of the child; he says "I am not willing to submit to that; I am willing to trust to you to treat the case." I immediately punctured that tumor, and put a piece of cotton, saturated with the arsenic in the tumor, and I says come back day after tomorrow. I repeated the same operation. Before three days the child came into the office and the tumor was gone. She had lost it while at play. She felt something loose in her mouth, and spit, and it came out. The mother brought the child up, and I examined the mouth, and there was a clean hole. I could see the little grinders, the two bicuspids down at the bottom. The disease had disappeared. I called in the doctors, and the surgeon examined it, and said: "That is the most perfect piece of work I have ever seen done." Not a sign of diseased tissue left. He says, "Wasn't you afraid to do that?" I says, "No." I washed the cavity out with a little carbolic acid wash, and in three weeks it was healthy and in perfect condition. When the girl was eighteen years old, she had as pretty a set of teeth as I ever saw. Had I gone entirely on the opinion of the surgeon I would have done differently. I was young in the business, and it was just a future for me. I had used arsenic only in a very few cases previously.

Dr. J. R. Beach: I think oral surgery is a good thing when needed. When it comes to criticising our reputation as a profession, I believe the whole world recognizes the value of the dental profession, and the whole world also recognizes the fact that we do not have to go outside our special field to get a reputation. The suggestion that presents itself to me, is that I do not believe we are adequately judged by our patients. Why should we give our whole time and attention to surgery when our duty is to do the general work of our profession, and when surgery is not our particular field.

Dr. J. A. Gardner in closing the discussion: I have enjoyed the discussion very much. I believe we all get more out of a fair discussion than we do from the formal paper.

Dr. Neal made a statement as to cancer noma. I think this is not a growth, but more of the nature of a decomposing or breaking down tissue. We know very little of it. We do not know that any one had ever been treated successfully by an operation.

One point I was very anxious should be brought out in the discussion was the necessity of teaching oral surgery in our schools. We must acknowledge the possibilities in oral surgery and our ability as dentists, and we must look to the dental profession to make adequate progress in oral surgery. We have just as bright young men coming into dental surgery as into medicine. How to teach oral surgery, to dental students and not have to rely on the medical men—to teach that branch is an important matter for our faculties to discuss.

Dr. Wood's remarks were very gratifying indeed, and I thank him for the good things he said.

He spoke of a case of necrosis. I believe in fitting the dentist for operative surgery. I do not believe in a dentist going beyond his field. To remove the tonsils or adenoids, I do not believe in that. I believe in confining the work to the teeth and jaws. With the necessary instructions, the dentist ought to be able to improve himself and his profession, if he is not an incompetent. We only get recognition for what we have accomplished. At present we have not a great field. We have a small field, and we can be masters of that. I was called upon a few days ago to treat a case with a very prominent surgeon of the South, who had been treating the case, and had been waiting for the development for an operation, while pus had been discharging for some little time. The teeth seemed to be all right, perfectly sound, and he had waited two years, and finally advised an operation for the resection of that jaw. He said that it was necessary. I say that a man is a criminal to perform surgery unless he feels fit to do it. Very few of us can diagnose the case. We are behind; we want to get busy. Surgical dentistry is what we want to work up to, but we shall have to do a lot of work to get up to that point.

We are most inefficient for the practice of dentistry, because we will not push through the margin of our pathological work. We are very inefficient physicians, because we do not impress upon dentists the importance of certain plans. I say that if we only do the work upon the teeth or practice in that alone, we will not be called upon for even that very much. I do not mean to leave the impression that the mechanical side is not practical, it does not deal with the problems of life and death. It does not deal in any of the vital sides of life. I maintain that we should prepare ourselves and the coming generation of dentists to take up everything in the way of oral surgery. We are well trained in the college, and we should do more and be able to handle a portion in oral surgery, not as teachers, but as operatives. We should so develop in the knowledge of surgery, that we will not have to refer our patients to the M.D.'s, because we have not a D. D. S. competent to do the work. I make that statement, so that we may profit by it. We have some bad surgical cases, and we should be able to take care of them. I just had a talk with Dr. Moore about the four years required to get thaft M. D. degree, which impressed me that the time in which to acquire that degree would give the profession the proper training. By all means get that M. D. degree. You have got to have it. The only men who are doing good oral surgery are medical graduates.

